# Transformation of Two Cases of Lung Adenocarcinoma into Pulmonary Sarcomatoid Carcinoma following Treatment

**DOI:** 10.1155/2021/6661772

**Published:** 2021-01-02

**Authors:** Zhenyu Yang, Yinfang Shen, Junhong Jiang, Ziyi Liu, Chuanyong Mu

**Affiliations:** ^1^Department of Respiratory and Critical Care Medicine, First Affiliated Hospital of Soochow University, Suzhou, China; ^2^Department of Pathology, First Affiliated Hospital of Soochow University, Suzhou, China

## Abstract

Accumulating evidence shows that histologic transformation is involved in the drug resistance of lung cancer. Moreover, it is common for lung adenocarcinoma to transform into small-cell lung cancer or squamous cell carcinoma; however, clinical cases with sarcomatoid transformation have been rarely reported. Thus, both the diagnosis and treatment of lung adenocarcinoma with sarcomatoid transformation remain difficult. Here, we discuss two patients with lung adenocarcinoma with sarcomatoid transformation—analyzing the diagnosis, clinical features, immunohistochemical characteristics, therapy, and prognosis—with the hope that this report will be used as a reference for future treatment of these patients.

## 1. Introduction

Lung cancer is the leading cause of cancer-related death worldwide [1]. In contrast with chemotherapy, targeted therapy constitutes a new route for the treatment of lung cancer. Epidermal growth factor receptor (EGFR) tyrosine kinase inhibitors (EGFR-TKIs) have shown a significant therapeutic effect in advanced non-small-cell lung cancer (NSCLC) with EGFR mutations; however, many patients may develop drug resistance due to various mechanisms of resistance, such as histologic transformation [2]. Histologic transformation of lung adenocarcinoma can be small-cell lung cancer, squamous cell carcinoma, or sarcomatoid carcinoma although clinical cases with sarcomatoid transformation are more uncommonly reported than those characterized by the former two conditions [3]. Here, we describe two patients with sarcomatoid transformation of lung adenocarcinoma to improve our understanding of this disease.

## 2. Case Report

### 2.1. Case 1

**Figure 1:**
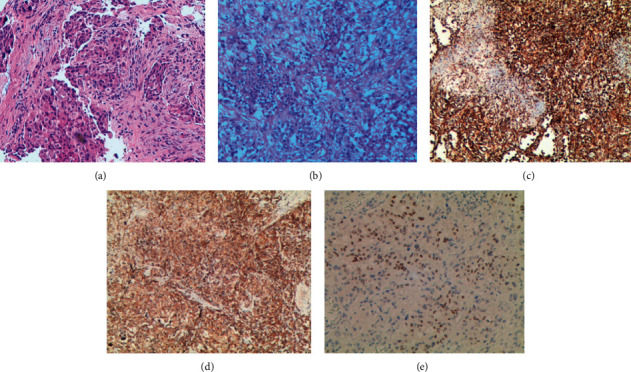
Pathological image of the patient diagnosed with lung adenocarcinoma in case 1 ((a) hematoxylin and eosin ×100). Pathological image of the patient diagnosed with PSC in case 1 ((b) pleomorphic carcinoma; hematoxylin and eosin ×100). Immunohistochemical images of the patient diagnosed with PSC in case 1 ((c) vimentin (+); (d) CK (+); (e) TTF-1 (+) taken using the Roche Ventana BenchMark XT autostainer (×100) (Roche Holding, Basel, Switzerland)).

A 46-year-old male patient was referred to our hospital on December 19, 2017, due to a cough. A chest computed tomography (CT) scan showed a mass on the apex of the right lung with swollen lymph nodes in the right hilar and mediastinum. Bronchoscope biopsy revealed adenocarcinoma (Figure 1(a)) and immunohistochemistry confirmed positive findings for cytokeratin (CK), thyroid transcription factor-1 (TTF-1), and napsin-A but a negative result for anaplastic lymphoma kinase (ALK). An EGFR mutation was not revealed with further genetic testing. Starting on January 12, 2018, the patient began receiving first-line chemotherapy consisting of pemetrexed plus carboplatin for four cycles, with a partial response. Disease progression occurred about nine months after the completion of chemotherapy. Since January 17, 2019, this patient has been treated with anlotinib (12 mg/day) and radiation therapy, experiencing progression-free survival for five months. A repeat chest CT scan was performed on July 5, 2019, and showed that the mass was 70% larger than that on January 16, 2019, suggesting further progression of the disease. Right pneumonectomy was performed on July 10, 2019, and the pathology and immunohistochemistry revealed lung adenocarcinoma with sarcomatoid transformation (pleomorphic carcinoma), with a sarcomatoid component that accounted for about 50% (Figure 1(b)), and showed positive results for vimentin, CK, CK7, and TTF-1, with the expression of TTF-1 being low (Figures 1(c), 1(d), and 1(e)). Regretfully, further programmed death-ligand 1 (PD-L1) detection and genetic testing for surgical specimens were not performed at this time due to the personal request of the patient's family. The patient subsequently continued to receive anlotinib and radiation therapy until he died from disease progression on December 10, 2019. However, significant expression of PD-L1 (80%) was found during supplementary PD-L1 detection of the remaining surgical specimens with the consent of the patient's family on November 20, 2020.

### 2.2. Case 2

**Figure 2:**
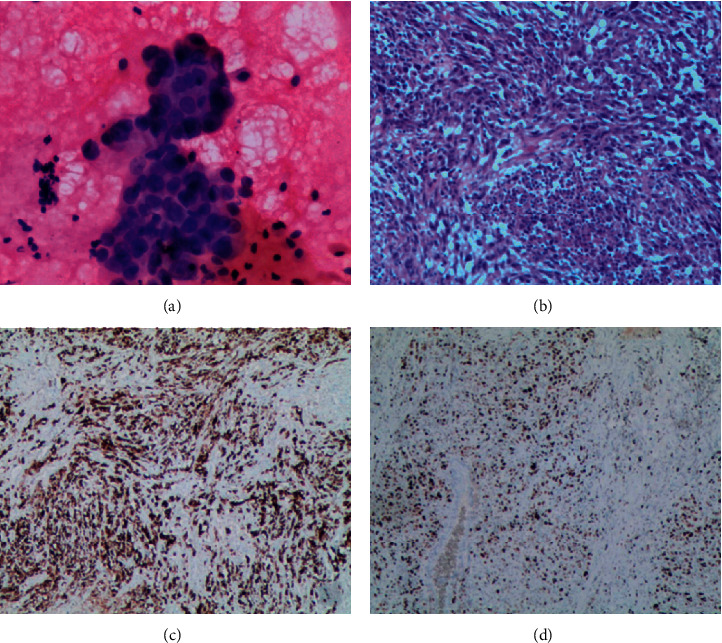
Pathological image of the patient diagnosed with lung adenocarcinoma in case 2 ((a) hematoxylin and eosin ×400). Pathological image of the patient diagnosed with PSC in case 2 ((b) pleomorphic carcinoma; hematoxylin and eosin ×100). Immunohistochemical images of the patient diagnosed with PSC in case 2 ((c) vimentin (+); (d) CK (+) taken using the Roche Ventana BenchMark XT autostainer (×100) (Roche Holding, Basel, Switzerland)).

A 53-year-old male patient presented to the hospital with cough and expectoration of phlegm. A chest CT scan was performed on December 1, 2017, and found a mass on the upper lobe of the left lung with swollen lymph nodes in both the hilar and mediastinum. This patient was subsequently diagnosed with lung adenocarcinoma following a bronchoscope brushing cytology (Figure 2(a)). From December 7, 2017, this patient received first-line chemotherapy consisting of pemetrexed and carboplatin for three cycles, resulting in stable disease. This patient underwent another bronchoscope biopsy for molecular testing on January 24, 2018, and revealed ALK positivity. He received seven more months of treatment with crizotinib (500 mg/day) until a chest and head CT scan revealed that the mass was 30% larger, with intracranial metastasis. Owing to his progressive disease, the patient received targeted therapy of alectinib (1,200 mg/day) and cranial radiotherapy, resulting in stable disease for nine months. However, a repeat chest CT scan was performed on July 1, 2019, and revealed that the mass was more than 30% larger than before. At this point, the patient failed to respond to albumin-bound paclitaxel and the disease continued to progress. From August 29, 2019, he received immunotherapy with PD-L1 for eight cycles and showed progression-free survival for four months. However, a chest CT scan performed on December 26, 2019, showed that the mass was 30% larger than before. The patient next underwent lobectomy of the left upper lung on January 20, 2020; at this stage, the pathology and immunohistochemistry suggested pleomorphic carcinoma, where the sarcomatoid component accounted for more than 80% (Figure 2(b)), and a weakly positive result for CK and CK18 and aberrant expression of vimentin, ALK, and PD-L1 (80%) (Figures 2(c) and 2(d)). The patient continued to receive targeted therapy with alectinib and immunotherapy with PD-L1 until April 9, 2020, but eventually succumbed to progressive disease on April 22, 2020.

## 3. Discussion

Advanced NSCLC with EGFR or ALK mutations respond well to related TKIs; however, drug resistance can occur due to various mechanisms such as drug-resistant mutations, activation of the bypass pathways, and histologic transformation. It has been reported that lung adenocarcinoma may transform into small-cell lung cancer or squamous cell carcinoma more frequently than into pulmonary sarcomatoid carcinoma (PSC) [2, 3].

PSC is a type of poorly differentiated NSCLC with an incidence of less than 0.1% that is found especially often in male smokers. According to the World Health Organization, PSC is defined as NSCLC with a sarcomatoid component such as pulmonary blastoma, carcinosarcoma, spindle-cell carcinoma, giant-cell carcinoma, or pleomorphic carcinoma [4]. The clinical features and imaging manifestations of PSC are nonspecific, making the diagnosis of PSC to be a challenging tasking. Biopsy alone cannot be enough to confirm the diagnosis of PSC unless combined with a pathological examination and immunohistochemistry analysis of the surgical specimens [5]. In our study, both patients were originally diagnosed with lung adenocarcinoma by bronchoscopy biopsy or bronchoscope brushing cytology but were later diagnosed with PSC by pathology and immunohistochemistry analysis of surgical specimens following disease progression.

The occurrence of lung adenocarcinoma with sarcomatoid transformation relies on epithelial-mesenchymal transition (EMT). Recent studies have shown that some biomarkers, including carcinoembryonic antigen (CEA), CK, CK7, TTF-1, and epithelial membrane antigen, are positively related with the epithelial component of PSC, while other markers such as vimentin and fascin are associated with the sarcomatoid component [6]. Besides, the typical characteristics of immunohistochemistry of PSC include the lack or absence of CK, CK7, and TTF-1 and the increased expression of vimentin. Some new studies have found that hepatocyte growth factor receptor gene (MET) mutations and high expression levels of PD-L1 are also indicators in the diagnosis and treatment of lung adenocarcinoma with sarcomatoid transformation [7]. When comparing the immunohistochemical results of the patient in case 1 between before and after sarcomatoid transformation, the expression of TTF-1 was decreased, but the vimentin expression was positive. The patient in case 2 was initially diagnosed with lung adenocarcinoma without immunohistochemical examination; meanwhile, when PSC was diagnosed, the immunohistochemical examination suggested a weakly positive CK result, a negative result for TTF-1, and aberrant expression of vimentin. All of the above results show the process of EMT.

Surgery is the main method to treat PSC, yet PSC has a high rate of recurrence and low overall survival rate. Furthermore, advanced PSC is highly resistant to chemotherapy [8, 9]. Targeted therapy and immunotherapy provide new options for the management of PSC. However, patients with sarcomatoid transformation often have lost the opportunity for surgery or have developed resistance to the available targeted drugs. Hsieh et al. [7] reported that lung adenocarcinoma with sarcomatoid transformation may be present in a high proportion of cases with aberrant MET activation and PD-L1 expression. These findings suggest that MET mutation or high expression of PD-L1 may occur in lung adenocarcinoma with sarcomatoid transformation, but the relationship still needs to be confirmed with further improved trials. Meanwhile, MET and PD-L1 inhibitors offer the possibility to manage these patients despite their drug resistance, whose potential also needs to be further studied. In our study, both patients showed high expression levels of PD-L1 when PSC was diagnosed, but the effect of targeted therapy or immunotherapy in these two patients was not satisfactory following the diagnosis of PSC given the rapid progression.

The process of EMT mediates the drug resistance of lung adenocarcinoma with sarcomatoid transformation [2]. Moreover, this occurs in the natural progression of tumors, drug resistance, and immune escape [10]. Therefore, the inhibition of EMT offers a potential treatment route in lung adenocarcinoma with sarcomatoid transformation. Twist1 is a helix-loop-helix transcription factor originally known to play a crucial role in embryonic development; however, it has been discovered that it may also induce the process of EMT and promote proliferation, differentiation, and metastasis in a wide variety of tumors [11, 12]. Liu et al. [13] reported that Twist1 was related to the stage and metastasis of tumors and suggested it could be a new target in patients with PSC. In their study, the expression of Twist1 in patients with PSC was higher than that in those with lung squamous cell carcinoma and patients with PSC who were Twist1-negative had better prognoses. An increasing number of studies have shown that the combination of anti-EMT therapy, chemotherapy, and immunotherapy may optimize the level of therapeutic efficacy in patients with PSC, but the effects of specific anti-EMT strategies, such as reversing the process of EMT or transforming the tumor cell directly, require more research [14].

## 4. Conclusion

In summary, PSC is a special pathologic type of NSCLC with a poor prognosis. Lung adenocarcinoma with sarcomatoid transformation increases the complexity of both the diagnosis and treatment. Pathology and immunohistochemistry of surgical specimens are required for the diagnosis of PSC. New gene mutations and immunotherapy after drug resistance may offer a novel choice for patients with lung adenocarcinoma with sarcomatoid transformation, while targeted inhibition of EMT-related targets provides a theoretical basis for anti-EMT therapy. However, numerous considerations including the type of gene mutations after drug resistance, the efficacy and safety of targeted therapy or immunotherapy, and the feasibility of anti-EMT drugs need to be explored in more basic and clinical trials.

## Data Availability

The data used to support this study are included within the article.
